# P-24. Seven Versus Fourteen Days of Antibiotics for Gram-Negative Bacteremia from a Urinary Tract Source:A Systematic Review and Meta-Analysis

**DOI:** 10.1093/ofid/ofaf695.254

**Published:** 2026-01-11

**Authors:** Graciela Luna, Andrea rodriguez Jimenez, Chidozie igbonagwam, Zein Barakat, Zoya Khan, Daniela Carralero Somoza, Garrett Lee Snyder, Imad Dibo, Mark Soliman, michael sabina

**Affiliations:** lakeland regional health medical center, Lakeland, FL; lakeland regional health medical center, Lakeland, FL; Lakeland Regional Health Medical Center, Lakeland, Florida; lakeland regional health medical center, Lakeland, FL; Lakeland Regional Health, Lakeland, Florida; lakeland regional health medical center, Lakeland, FL; lakeland regional health medical center, Lakeland, FL; lakeland regional health medical center, Lakeland, FL; Lakeland Regional Health, Lakeland, Florida; Iakeland regional health, lakeland, Florida

## Abstract

**Background:**

The optimal duration of antibiotic therapy for gram-negative bacteremia sourced from urinary tract infections (UTI) remains uncertain. We performed a systematic review and meta-analysis comparing short-course (approximately 7 days) versus prolonged-course (approximately 14 days) antibiotic therapy in this population.

**Figure 1:**
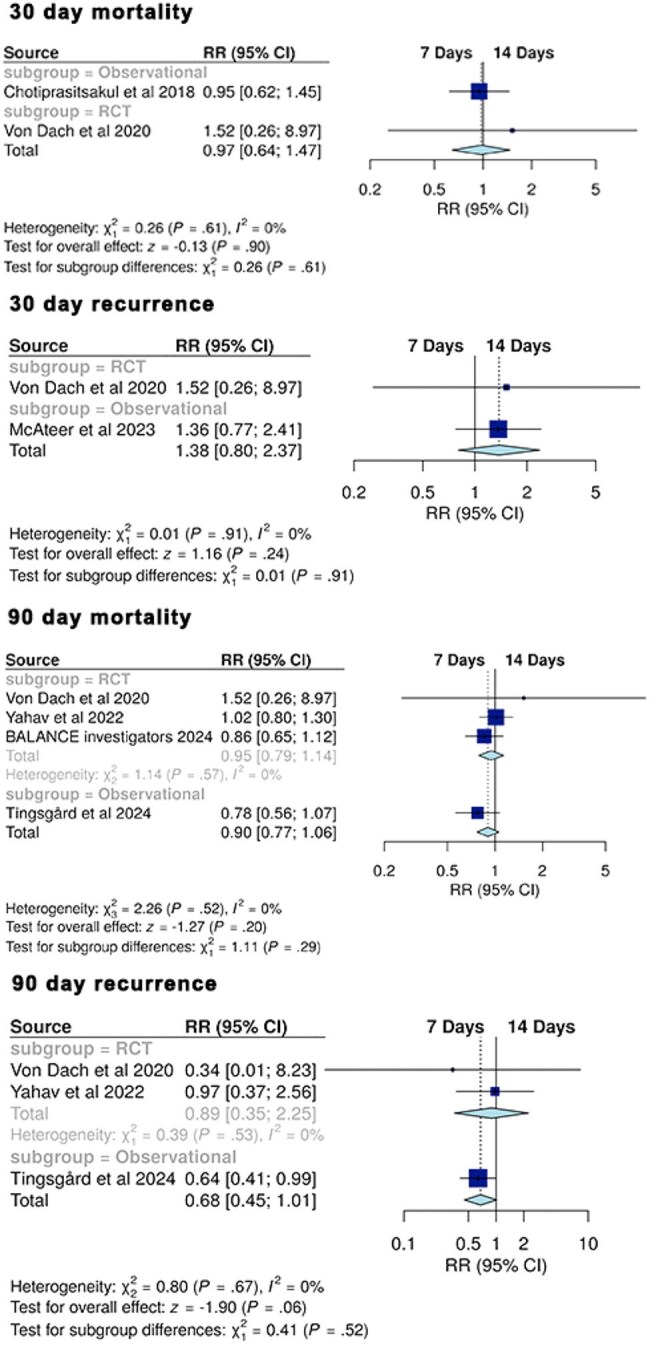
Forest plot of outcomes

**Table 1:**
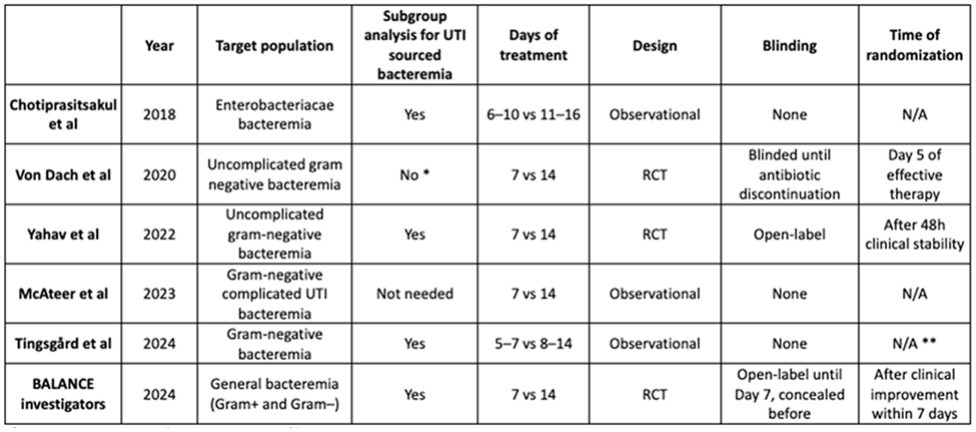
Study design

**Methods:**

We systematically searched PubMed, Embase, and ClinicalTrials.gov through April 26, 2025. Studies were included if they compared 7-day versus 14-day antibiotic therapy in gram-negative bacteremia with ≥65% UTI source or performed a dedicated UTI subgroup analysis. Outcomes assessed included 30- and 90-day mortality and recurrence rates. Risk ratios (RR) were pooled using a random-effects model. Noninferiority was assessed using a prespecified margin of RR 1.25, and superiority was assessed with a threshold of RR < 1.00.

**Results:**

Six studies (three randomized trials, three observational cohorts) encompassing 4,448 patients were included. There were no significant differences between short- and prolonged-course therapy for 30-day mortality (RR 0.97, 95% CI 0.64–1.47; p=0.90), 30-day recurrence (RR 1.38, 95% CI 0.80–2.37; p=0.24), 90-day mortality (RR 0.90, 95% CI 0.77–1.06; p=0.20), or 90-day recurrence (RR 0.68, 95% CI 0.45–1.01; p=0.06).

**Conclusion:**

Our findings suggest that a 7-day course may be sufficient for most patients with UTI-sourced gram-negative bacteremia.

**Disclosures:**

All Authors: No reported disclosures

